# Association of methylenetetrahydrofolate reductase gene polymorphisms and maternal folic acid use with the risk of congenital heart disease

**DOI:** 10.3389/fped.2022.939119

**Published:** 2022-09-08

**Authors:** Taowei Zhong, Xinli Song, Yiping Liu, Mengting Sun, Senmao Zhang, Letao Chen, Jingyi Diao, Jinqi Li, Yihuan Li, Jing Shu, Jianhui Wei, Ping Zhu, Tingting Wang, Jiabi Qin

**Affiliations:** ^1^Department of Epidemiology and Health Statistics, Xiangya School of Public Health, Central South University, Changsha, China; ^2^Guangdong Cardiovascular Institute, Guangdong Provincial People's Hospital, Guangdong Academy of Medical Sciences, Guangzhou, China; ^3^National Health Council (NHC) Key Laboratory of Birth Defect for Research and Prevention, Hunan Provincial Maternal and Child Health Care Hospital, Changsha, China

**Keywords:** congenital heart disease, methylenetetrahydrofolate reductase gene, periconceptional folate supplementation, interaction effects, case-control study

## Abstract

**Background:**

To systematically evaluate the association of *MTHFR* genetic polymorphisms, maternal folic acid intake, and the time when folic acid intake was started with the risk of congenital heart disease (CHD) and investigated the role of their interaction on infant CHD risk in Chinese populations.

**Methods:**

A case–control study involving 592 CHD cases, 617 health controls, and their mothers was performed. The exposures of interest were single nucleotide polymorphisms (SNPs) of the *MTHFR* gene, maternal folic acid use, and the time when folic acid use was started. We applied the logistic regression model to explore the strength of association.

**Results:**

Our findings showed that mothers lacking folic acid intake had a significantly higher risk of CHD in offspring (aOR = 2.00; 95%CI: 1.34–2.98). Mothers who started to use folic acid from the first trimester of the fetation (aOR = 1.65; 95% CI: 1.22–2.23) or from the second trimester of the fetation (aOR = 7.77; 95% CI: 2.52–23.96), compared with those starting to use folic acid from 3 months previous to the conception, were at a significantly higher risk of CHD in offspring. Genetic variants at rs2066470 (AA vs. GG: aOR = 5.09, 95%CI: 1.99–13.03), rs1801133 (AA vs. GG: aOR = 2.49, 95%CI: 1.58–3.93), and rs1801131 (TG vs. TT: aOR = 1.84, 95%CI: 1.36–2.50; GG vs. TT: aOR = 3.58, 95%CI: 1.68–7.63) were significantly associated with the risk of CHD based on the multivariate analysis. Additionally, statistically significant interactions between maternal folic acid intake and genetic variants of the *MTHFR* gene at rs1801133 and rs1801131 were observed.

**Conclusion:**

An association of maternal folic acid intake and the time when intake was started with the risk of CHD in offspring was found. What's more, maternal folic acid fortification may help counteract partial of the risks of CHD in offspring attributable to *MTHFR* genetic mutations.

**Registration number:**

http://www.chictr.org.cn/edit.aspx?pid=28300&htm=4, identifier: ChiCTR1800016635.

## Introduction

Congenital heart disease (CHD) is caused by abnormal cardiac development during the embryonic period, which refers to a cluster of structural heart and vascular defects at birth. It is a primary common cause of the congenital anomaly that led to disability and death in infancy and is responsible for one-third of all major congenital malformations, afflicting ~1% of live birth and birthing the 1.35 million infants with CHD each year worldwide (1, 2). Considering the complexity of embryonic heart development, the pathogenesis of the majority of CHD remains unclear.

Previous studies have indicated that fortified folic acid was significantly related to the reduced risk of neural tube defects, orofacial defects, and CHD (3–6). However, the underlying mechanisms by which folate supplementation reduces the risk of CHD were not fully elucidated. It is plausible that genetic polymorphisms related to folate utilization may help to bring out the undefined mechanism of cardiac development. The 5,10-methylenetetrahydrofolate reductase (*MTHFR*) gene is frequently involved in the etiology of CHD. The *MTHFR*, encoded by the *MTHFR* gene, takes a part in the folate metabolism and catalyzes the conversion of 5,10-methylenetetrahydrofolate to 5-methyltetrahydrofolate (7), which generates a methyl donor that played a key role in the conversion of homocysteine to methionine (8). Above all, the *MTHFR* gene involves the folate-homocysteine cycle and its mutations inhibit the elimination of homocysteine, which is an independent risk factor for the occurrence of CHD (9, 10). For instance, the genetic polymorphisms rs1801133 (C667T) and rs1801131 (A1298C) of the *MTHFR* gene piqued interest. The rs1801133 *MTHFR* polymorphism affected the optimal activity of the enzyme (11) and increased the level of homocysteine (8) and the other variation (*MTHFR* A1298C) within the presumed regulatory domine also inhibited the enzyme activity (12). It is worth noting that findings from previous studies involved in the association of the two genetic mutations with the risk of CHD were conflicting (13, 14). Additionally, previous studies focused mainly on the above-mentioned loci and have ignored the other *MTHFR* gene loci. Meanwhile, these studies did not assess whether other *MTHFR* gene mutations and maternal folic acid intake were associated with the risk of CHD.

Hence a hospital-based case–control study based on the Han Chinese population was performed to explore the association of multiple genetic variants of the *MTHFR* gene, maternal folic acid use, the time when folic acid use started, and their interactions with the occurrence of CHD in offspring, which will help to extend prevention, improve the primary prevention strategies involved risk genetic variations, and provide new loci which may help define the underlying mechanisms by which folate impacts on the CHD risk.

## Subjects and methods

### Study design and recruitment of the study

Exposures of interest were maternal folic acid use during this pregnancy, defined as a minimum of 0.4 mg of folic acid daily for > 5 days per week regularly during 3 months before conception or the first trimester of pregnancy (4), the time when folic acid intake started (i.e., 3 months prior to conception, the first trimester of pregnancy, or second trimester of pregnancy) and *MTHFR* genetic polymorphisms. The Outcome of interest was non-syndromic CHD.

In this, we conducted a case–control study. From November 2017 to January 2020, the recruitment of the case–control study was performed in the Hunan Provincial Children's Hospital, Changsha, Hunan Province, China. Two different apartments in the same hospital performed the present study, one was the Department of Cardiothoracic Surgery that provided cases and the other was the Department of Child Healthcare that recruited controls during the same study period as the cases. Eligible participants in this hospital were conducted health counseling or a medical examination. Children afflicted with CHD and their mothers were included in the case group. As a control, children diagnosed with no occurrence of any congenital deformities after medical examination and their mothers were recruited into the control group. We required that all cases and controls were recruited when children were younger than 1 year to minimize the recall bias of exposure during the pre-pregnancy to the early stage of this pregnancy. Considering potential confounding factors from genetic and cultural differences related to the ethnic background, all eligible participants were of Han Chinese descent. Additionally, eligible participants were interviewed face-to-face in the same way to collect the personal information and professionally trained investigators performed the interview for the quality of a questionnaire. All participants signed informed consent, belong to singleton pregnancies for this pregnancy, and provided the blood sample. However, we excluded cases with syndromic CHDs and structural malformations involving another organ system or known chromosomal abnormalities. The children whose mothers conceived this time by assisted reproductive technology including *in vitro* fertilization (IVF) and intracytoplasmic sperm injection (ICSI) were excluded. Mothers who had reported or were diagnosed with depression or a psychiatric illness were excluded.

### Information collection

We also collected information by a standardized questionnaire as follows: child's gender, maternal demographic characteristics (i.e., age at the time of this pregnancy, education status, and residence location), family history (i.e., consanguineous marriages), adverse pregnancy history before this pregnancy (i.e., adverse birth outcomes and pregnancy-related complications), personal lifestyle during the periconceptional period (i.e., active smoking, passive smoking, and drinking alcohol), and history of exposure to environmentally hazardous substances in the periconceptional period (i.e., harmful chemicals). In China, each pregnant woman is given a perinatal health care handbook (PHCH) that recorded pregnancy or personal information by a medical professional or the woman, including the basic demographic characteristics, personal habits, family history, folic acid fortification, medical examination results, and medical history during this pregnancy. We confirmed the correspondence of information from the questionnaire with PHCH to control the accuracy of the questionnaire. Face-to-face investigations and PHCH, considered the most trustworthy information source, were the source of relevant information, which could help to collect information accurately and integrally.

### Sequencing of *MTHFR* gene SNPs

A previous study expounded on the selection method of candidate loci of the *MTHFR* gene (15). SNPBrowser™ program (version 3.0) provided by Applied Biosystems Inc. could help select SNP markers from the public database-HapMap database. Based on the criteria, tagging SNPs with the pairwise *r*^2^ ≤ 0.8 and minor allele frequencies (MAF) <10% were excluded. In the end, the loci rs2274976, rs4846052, rs7525338, rs1476413, rs1801133, rs4846051, rs2066470, and rs1801131 of the *MTHFR* gene, all within the coding region, were included as candidate loci in the present study.

Genotyping was performed by 3 ml of peripheral venous blood from children. EDTA-treated (ethylenediaminetetraacetic acid) anticoagulant tubes reserved blood samples provisionally, which were then centrifuged at 1,300 g for 15 min. After centrifuging blood samples, blood cells were separated and preserved at −80°C before genotyping was conducted. Based on the manufacturer's standard protocol, blood cells extracted genomic DNA, dissolved in sterile TBE (Tris-borate-EDTA) buffer, which was applied to the QIAamp DNA Mini Kit (Qiagen, Valencia, CA, USA). Ultraviolet spectrophotometry detected the concentration and purity of the DNA solution, which could help detect whether the DNA was appropriate as a template for polymerase chain reaction (PCR). A matrix-assisted laser desorption and ionization time-of-flight mass spectrometry MassARRAY system (Agena iPLEX assay, San Diego, CA, USA) tested genetic polymorphisms of the *MTHFR* gene. We required that the error rate of genotyping ≤ 5% and the sectionalization of the control and case was blinded to the experimenters performing the genotyping.

### Statistical analysis

In the study, absolute numbers and percentages described the qualitative data, and its comparisons between two groups applied the Pearson chi-square test or Fisher's exact test. For the ordinal categorical variable, the Wilcoxon rank-sum test was applied to calculate the difference across the two groups. The Hardy–Weinberg equilibrium (HWE) was gauged by genotype frequencies of the *MTHFR* gene in the control group. Based on the two-phase analytical method from genetic model selection (GMS), we assessed the association of *MTHFR* genetic polymorphisms with CHD risk, and the previous study described the calculation process (16). According to the *Z*_HWDTT_ statistic with C = 1.645, the genetic model of SNPs could be identified (the genetic model was decided as the dominant model if *Z*_HWDTT_ ≤ C, recessive model if *Z*_HWDTT_ > C, additive model if *Z*_HWDTT_ ≥ C or *Z*_HWDTT_ < C). Odds ratios (OR) and their 95% confidence intervals (95% CI) calculated by the logistic regression analysis were applied to evaluate the strength of associations. Considering confounding factors, adjusted ORs (OR_adj_), obtained from multivariable logistic regression, were used to evaluate the independent risk factors of CHD. Considering the potential confounders on the interaction term, we entered the covariate-by-environment and the covariate-by-gene interaction terms in the same model when assessing the gene-environment (G × E) interactions (17). All tests were conducted at a *P-*value < 0.05 with a two-sided approach and those results with FDR-*P-*value < 0.01 was statistically significant. What's more, to minimize the type I error brought from the multiple testing, we used the false discovery rate (FDR-*P*) to correct it. All statistical analyses were conducted by SAS 9.1 (SAS Institute, Cary, NC, USA).

## Results

### Participants baseline characteristics

From November 2017 to January 2020, we recruited a total of 592 mothers and their CHD infants into the case group and 617 mothers and their healthy infants into the control group. A total of 132 (10.9%) cases were diagnosed with atrial septal defects, 434 (35.9%) cases were diagnosed with ventricular septal defects, 25 (2.0%) were diagnosed with atrioventricular septal defects, 163 (13.1%) were diagnosed with patent ductus arteriosus, 32 (2.6%) with tetralogy of Fallot, 7 (0.6%) with aortopulmonary windows, and 3 (0.2%) with complete transposition of great arteries. Notably, some cases were diagnosed with multiple CHD subcategories simultaneously, so the total amount of the various subcategories was not equal to 592. Table 1 illustrated comparisons of baseline characteristics across two groups, and Supplementary Table S1 stated comparisons of baseline characteristics based on the use status of folic acid supplement in the control group. Overall, we found a significant difference across the two groups in the following factors: infant gender, maternal education status, resident location, family history (consanguineous marriages), adverse pregnancy history (induced abortion or labor, fetal death or stillbirth, neonatal death, hypertension of pregnancy, and gestational diabetes mellitus), personal lifestyle in the 3 months before this pregnancy (active smoking, positive smoking, and drinking alcohol), and exposure to environmental risk factors in the 3 months before this pregnancy (harmful chemicals). As shown in Supplementary Table S1, all of the covariates were evenly distributed in the groups, which may prove the less interference of confounding factors on the results and the robustness of the association. The significant factors with *P* < 0.05 were adjusted when performing the multivariable logistic regression.

**Table 1:** Comparison of baseline characteristics in case and control groups.

**Variables**	**Control group** **(*n* = 617)**	**Case group** **(*n* = 592)**	**Univariable analysis**
**Demographic characteristics**			
Gender			*χ^2^* = 2.513, *P* = 0.000
Male	405 (65.6%)	303 (51.2%)	
Female	212 (34.4%)	289 (48.8%)	
Maternal age at pregnancy onset (≤ 35)	554 (89.8%)	523 (88.3%)	*χ^2^* = 0.648, *P* = 0.421
**Maternal education status**			
Less than primary or primary	7 (1.1%)	87 (14.0%)	*Z* = −0.353, *P* = 0.000^*^
Junior high school	117 (18.9%)	263 (42.4%)	
Senior middle school	217 (35.0%)	167 (26.9%)	
College or above	279 (45.0%)	103 (16.6%)	
Residence location (rural areas)	339 (54.9%)	430 (72.6%)	*χ^2^* = 40.851, *P* = 0.000
**Family history**			
Consanguineous marriages (yes)	3 (0.5%)	19 (3.2%)	*χ^2^* = 12.541, *P* = 0.000
**Adverse pregnancy history before this pregnancy**			
Spontaneous abortion (yes)	60 (9.7%)	65 (11.0%)	*χ^2^* = 0.514, *P* = 0.474
Induced abortion or labor (yes)	189 (30.6%)	233 (39.4%)	*χ^2^* = 10.125, *P* = 0.001
Fetal death or stillbirth (yes)	2 (0.3%)	18 (3.0%)	*χ^2^* = 13.703, *P* = 0.000
Premature delivery (yes)	6 (1.0%)	9 (1.5%)	*χ^2^* = 0.740, *P* = 0.390
Low birth weight (yes)	3 (0.5%)	9 (1.5%)	*χ^2^* = 3.287, *P* = 0.070
Neonatal death (yes)	0	9 (1.5%)	*P* = 0.002^**^
Ectopic pregnancy (yes)	16 (2.6%)	13 (2.2%)	*χ^2^* = 0.204, *P* = 0.652
Congenital malformation (yes)	2 (0.3%)	7 (1.2%)	*χ^2^* = 3.012, *P* = 0.083
Hypertension of pregnancy (yes)	9 (1.5%)	33 (5.6%)	*χ^2^* = 15.261, *P* = 0.000
Gestational diabetes mellitus (yes)	17 (2.8%)	58 (9.8%)	*χ^2^* = 25.749, *P* = 0.000
**Personal lifestyle in the 3 months before this pregnancy**			
Active smoking	13 (2.1%)	24 (4.1%)	*χ^2^* = 3.861, *P* = 0.049
Positive smoking	227 (36.8%)	310 (52.4%)	*χ^2^* = 29.682, *P* = 0.000
Drinking alcohol	43 (7.0%)	73 (12.3%)	*χ^2^* = 10.014, *P* = 0.002
**Exposure to environmental risk factors in the 3 months before this pregnancy**			
Harmful chemicals	46 (7.5%)	112 (18.9%)	*χ^2^* = 34.947, *P* = 0.000

* Different between groups were tested by the Wilcoxon rank-sum test.

** Differences between groups were tested by the Fisher's exact test.

### Maternal folic acid intake and CHD risk in offspring

As Table 2 showed, the percentage of mothers with CHD infants who did not use folic acid was significantly higher than those of the control group (14.4 vs. 7.0%, *P* = 0.000). The present study showed mothers not using folic acid were significantly associated with a higher occurrence of CHD in offspring when compared with those using folic acid (aOR = 2.00; 95%CI: 1.34–2.98). Meanwhile, the time when folic acid intake started was related to the CHD risk. Mothers who started to use folic acid from the first trimester of the fetation (aOR = 1.65; 95% CI: 1.22–2.23) or from the second trimester of the fetation (aOR = 7.77; 95% CI: 2.52–23.96), compared with those starting to use folic acid from 3 months previous to the conception, were at a significantly higher risk of CHD in offspring.

**Table 2:** Association of maternal folic acid use with the risk of CHD.

**Maternal folic acid use**	**Control group (*n* = 617)**	**Case group** **(*n* = 592)**	**Univariable analysis**	**Unadjusted OR (95% CI)**	**Adjusted OR (95% CI)^†^**
**Use of folic acid for this pregnancy**					
Yes	574 (93.0%)	507 (85.6%)	*χ^2^* = 17.424, *P* = 0.000	1.00 (reference)	1.00 (reference)
No	43 (7.0%)	85 (14.4%)		2.24 (1.52–3.29)	2.00 (1.3–42.98)
Time of starting to use folic acid among women using folic acid				2.17 (1.66–2.84)	1.50 (1.08–2.09)
Three months prior to conception	162 (28.4%)	85 (16.8%)	*χ^2^* = 37.514, *P* = 0.000	1.00 (reference)	1.00 (reference)
First trimester of pregnancy	403 (70.7%)	393 (77.5%)		1.86 (1.38–2.50)	1.65 (1.22–2.23)
Second trimester of pregnancy	5 (0.9%)	29 (5.7%)		11.05(4.13–29.59)	7.77 (2.52–23.96)

CHD, congenital heart disease; CI, confidence interval; OR, odds ratio.

† Adjusted for gender, maternal age at pregnancy onset, education status, residence location, adverse pregnancy history (induced abortion or labor, fetal death or stillbirth, neonatal death, hypertension of pregnancy, gestational diabetes mellitus), family history (consanguineous marriages), maternal lifestyle before this pregnancy, harmful chemicals' exposure history in this pregnancy.

### Genotypes frequencies, HWE tests and GMS of SNPs at the *MTHFR* gene

Supplementary Table S2 displayed the allele frequencies and the *P*-value of the HWE test of the *MTHFR* gene. The frequencies of minor alleles for two loci (rs7525338 and rs4846051) were absent and excluded from further analyses. The HWE test suggested that the genotype frequencies of the *MTHFR* gene in the control group, shown in Supplementary Table S3, were consistent with HWE, which provided the good group representativeness of our sample.

The Hardy–Weinberg disequilibrium trend test was applied to calculate the *Z*_HWDTT_ statistic of every locus, whose results were shown in Supplementary Table S3 accordingly. The genetic models, emerging from the comparison of *Z*_HWDTT_ statistic with C = 1.645, exhibited as follows: The genetic models of SNPs including rs2274976, rs1476413, rs1801133, and rs1801131 were sorted into the additive model. The genetic models of rs4846052 and rs2066470 were sorted into the dominant model. The genetic models of overall SNPs at the *MTHFR* gene were applied to explore the correlation of SNPs with the CHD risk on the counterpart for the genetic model.

### *MTHFR* genetic polymorphisms and risk of CHD

**Figure 1:**
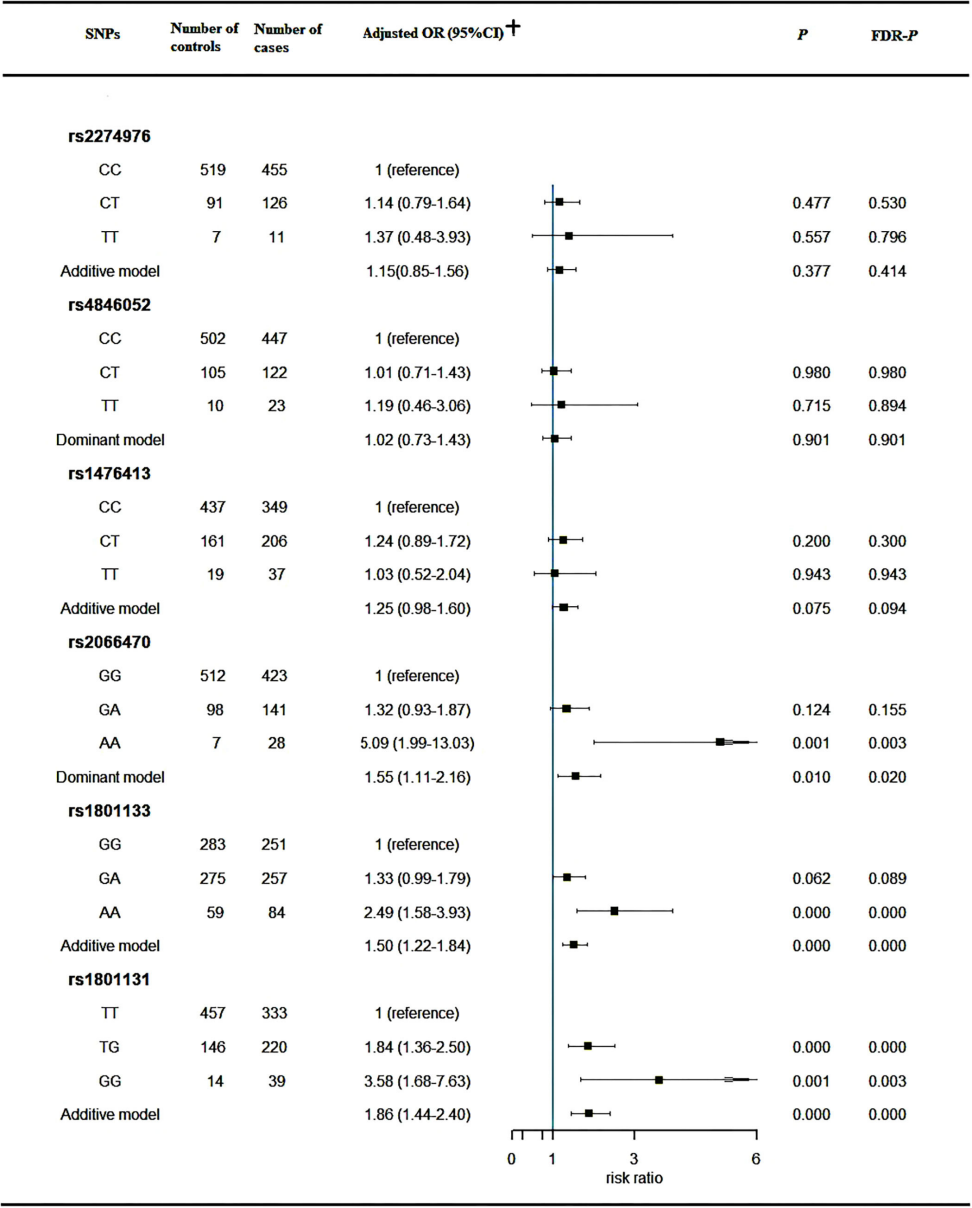
Association between *MTHFR* genetic variants and risk of CHD. CHD, congenital heart disease; CI, confidence interval; OR, odds ratio; *MTHFR*, Methylenetetrahydrofolate reductase; FDR, false discovery rate. ^†^Adjusted for gender, maternal education status, residence location, adverse pregnancy history (induced abortion or labor, fetal death or stillbirth, neonatal death, hypertension of pregnancy, gestational diabetes mellitus), family history (consanguineous marriages), maternal lifestyle before this pregnancy, harmful chemical exposure history in this pregnancy.

Genetic polymorphisms of the *MTHFR* gene related to the CHD risk were displayed in Figure 1. Our study found that the genetic variants at rs2066470 (AA vs. GG: aOR = 5.09, 95%CI: 1.99–13.03), rs1801133 (AA vs. GG: aOR = 2.49, 95%CI: 1.58–3.93; additive model: aOR: 1.50, 95%CI: 1.22–1.84), and rs1801131 (TG vs. TT: aOR = 1.84, 95%CI: 1.36–2.50; GG vs. TT: aOR = 3.58, 95%CI: 1.68–7.63; additive model: aOR: 1.86, 95%CI: 1.44–2.40), were significantly associated with the risk of CHD based on the multivariate analysis.

### Gene-environment interactions between the *MTHFR* gene and folic acid use

Gene-environment interactions between the *MTHFR* gene and folic acid fortification on the risk of CHD were summarized in Table 3. Our findings showed that statistically significant interactions between maternal folic acid intake and genetic mutants of the *MTHFR* gene at rs1801133 (aOR = 1.28, 95%CI: 1.08–1.62) and rs1801131 (aOR = 1.59, 95%CI: 1.28–1.98). We performed the crossover analysis to further assess the interaction effects of *MTHFR* SNPs and maternal folic acid on the occurrence of CHD (Table 4). Mothers who had been exposed to folic acid and whose children had the wild-type genotype simultaneously were the reference group. It was suggested that when compared with those in the reference group, mothers did not use folic acid, whose children, meanwhile, had variant genotypes at rs1801133 (aOR = 3.27, 95%CI = 1.85–5.78) and rs1801131 (aOR = 2.85, 95%CI: 1.29–6.29) had significant associations with increased risk of CHD in offspring.

**Table 3:** Multiplicative interaction between SNPs of the *MTHFR* gene and maternal folic acid use detected by logistic regression.

***MTHFR* genotype**	**Maternal folic acid use**		
	**Adjusted OR (95%CI)^†^**	***P***	**FDR*-P***
rs2274976	1.13 (0.89–1.43)	0.334	0.638
rs4846052	1.06 (0.84–1.34)	0.603	0.849
rs1476413	1.32 (1.07–1.63)	0.010	0.093
rs2066470	1.36 (1.07–1.73)	0.013	0.016
rs1801133	**1.28 (1.08–1.62)**	**0.011**	**0.003**
rs1801131	**1.59 (1.28–1.98)**	**0.000**	**0.000**

SNP, single nucleotide polymorphism; MTHFR, Methylenetetrahydrofolate reductase; CI, confidence interval; OR, odds ratio; FDR, false discovery rate.

† Adjusted for gender, maternal age at pregnancy onset, education status, residence location, adverse pregnancy history (induced abortion or labor, fetal death or stillbirth, neonatal death, hypertension of pregnancy, gestational diabetes mellitus), family history (consanguineous marriages), maternal lifestyle before this pregnancy, harmful chemicals' exposure history in this pregnancy.

Bold font indicates statistical significant.

**Table 4:** Crossover analysis in assessing the gene-environmental interaction between the *MTHFR* gene and maternal folic acid supplementation for risk of CHD.

***MTHFR* genotype**	**Maternal folic acid use**	**Number of controls**	**Number of cases**	**Adjusted OR (95%CI)^†^**	***P***	**FDR*-P***
**rs2274976**						
Wild genotype (CC)	Yes	489	409	1.00 (reference)		
Variant genotype (CT + TT)	Yes	88	117	1.29 (0.91–1.83)	0.157	0.235
Wild genotype (CC)	No	33	72	1.25 (0.76–2.06)	0.376	0.752
Variant genotype (CT + TT)	No	10	22	1.03 (0.42–2.49)	0.955	0.955
**rs4846052**						
Wild genotype (CC)	Yes	469	384	1.00 (reference)		
Variant genotype (CT + TT)	Yes	105	123	1.18 (0.84–1.65)	0.339	0.339
Wild genotype (CC)	No	33	63	1.13 (0.68–1.86)	0.643	0.772
Variant genotype (CT + TT)	No	10	22	1.36 (0.58–3.22)	0.481	0.577
**rs1476413**						
Wild genotype (CC)	Yes	404	304	1.00 (reference)		
Variant genotype (CT + TT)	Yes	170	203	1.37 (0.96–1.83)	0.030	0.060
Wild genotype (CC)	No	33	45	0.87 (0.51–1.63)	0.625	0.938
Variant genotype (CT + TT)	No	10	40	2.66 (1.21–5.85)	0.015	0.023
**rs2066470**						
Wild genotype (GG)	Yes	477	362	1.00 (reference)		
Variant genotype (GA + AA)	Yes	97	145	1.83 (1.32–2.54)	0.013	0.037
Wild genotype (GG)	No	35	61	2.42 (1.49–3.93)	0.007	0.021
Variant genotype (GA + AA)	No	8	24	3.48 (1.37–8.83)	0.009	0.027
**rs1801133**						
Wild genotype (GG)	Yes	263	220	1.00 (reference)		
Variant genotype (GA + AA)	Yes	311	287	1.21 (0.92–1.60)	0.170	0.204
Wild genotype (GG)	No	20	31	1.69 (0.85–3.35)	0.133	0.399
Variant genotype (GA + AA)	No	23	54	**3.27 (1.85–5.78)**	**0.000**	**0.000**
**rs1801131**						
Wild genotype (TT)	Yes	424	295	1.00 (reference)		
Variant genotype (TG + GG)	Yes	150	212	1.89 (1.39–2.58)	0.000	0.000
Wild genotype (TT)	No	33	38	0.97 (0.53–1.76)	0.910	0.999
Variant genotype (TG + GG)	No	10	47	**2.85 (1.29–6.29)**	**0.009**	**0.018**

CHD, congenital heart disease; CI, confidence interval; OR, odds ratio; FDR, false discovery rate; MTHFR, Methylenetetrahydrofolate reductase.

† Adjusted for gender, maternal age at pregnancy onset, education status, residence location, adverse pregnancy history (induced abortion or labor, fetal death or stillbirth, neonatal death, hypertension of pregnancy, gestational diabetes mellitus), family history (consanguineous marriages), maternal lifestyle before this pregnancy, harmful chemical exposure history in this pregnancy.

Bold font indicates statistical significant.

We also performed the stratified analysis of maternal folic acid use to assess the independent effect of *MTHFR* gene polymorphisms on the CHD risk (Supplementary Figure S1). Our findings suggested that the CHD risk in offspring with variant genotypes, whose mothers did not fortify folic acid was significantly increased when compared with those with variant genotypes whose mothers fortified folic acid. Among mothers not using folic acid, the CHD risk in children with variants genotypes was 4.72 (95%CI: 1.99–11.20) for rs1801131. However, among mothers fortifying folic acid, the risk for CHDs with variants genotypes at rs1801131 was 1.93 (95%CI: 1.45–2.59). Additionally, among mothers using folic acid, genetic mutation at rs2066470 (aOR = 1.72, 95%CI: 1.24–2.39) was significantly associated with CHD risk.

## Discussion

Conclusive evidence exists that folic acid, taken periconceptionally, is known to reduce about 40% risks of CHD, especially for the most severe CHD phenotypes (4), which makes it an appealing perspective to explore the relationship between genetic polymorphisms in folate metabolism and the CHD risk. The *MTHFR* gene, in which inherited mutations can influence an optically active enzyme and, accordingly, elevated homocysteine levels (18), functions in the folate-homocysteine metabolic pathway and has an impact on protein, DNA, and RNA synthesis. The enzyme, therefore, is in charge of maintaining the balance of folic and homocysteine to prevent dysfunction in cells (11). Population-based studies have demonstrated a sound association of *MTHFR* genetic variants with the risk of CHD (19, 20); however, results of previous studies on this topic have been dissident, with the majority restricted to a minor number of functional non-synonymous polymorphisms (21–23). Thus, the study aimed at probing the effect of other mutations of the *MTHFR* gene on the CHD risk in Han Chinese populations. Apart from that, what was explored mainly was the association of multiple genetic variants of the *MTHFR* gene, maternal folic acid use, the time when folic acid use started, and their interaction with CHD risk.

Our results indicated that the offspring of mothers who did not utilize folic acid for the current pregnancy had a significantly different risk of CHD compared with the offspring whose mother used folic acid, which was in line with previous studies on this tissue (3–5). The preventive status of folic acid has been entrenched for a long time. Considering the benefits of folic acid in preventing congenital malformations, the United States health authorities introduced mandatory folic acid fortification (24). To this day, folic acid intake during the period of preconception has emerged as a routine means of preventing neural tube defects (6), which dodges several nongenetic risk factors. The preventive effect of folic acid on CHD is gaining public perception. Some studies focused on the association of periconceptional folic acid with CHD, which emphasized the preventive role of folic acid (25–28). The folic acid supplement can not only affect the stage of embryonic development but can also protectively affect the period of pregnancy (preterm birth, gestational hypertension/preeclampsia, small for gestational age) (29–32). Folic acid is also conducive to adults (Alzheimer's disease, central nervous system) (33, 34) and improves the vascular function (35).

This study also indicated the significant difference between the time when starting to use folic acid and the CHD risk. The finding showed that when compared with mothers who started to use folic acid from 3 months of preconception, mothers starting to use folic acid from the first trimester or from the second trimester of gestation had a significantly increased CHD risk in offspring. Few studies considered the time points of starting to use folic acid when appraising the effect of folic acid on the occurrence of CHD. There is conclusive evidence that a folic acid supplement is recommended for all women capable of becoming pregnant during the periconceptional period to reduce a certain portion of CHD risks. However, the major problem is that there was a high percentage of women of reproductive age who had unanticipated pregnancies and used the folic acid irregularly, of which in the United States, Hungary, and so on were 50%, and they cannot able to take benefit from the primary preventive approach during the periconceptional period (36). It is disappointing that when the unplanned pregnancy is identified, already there is the beginning of the closing of the critical window for heart development, which renders the target of CHD prevention “abortive”. Compellent findings demonstrated that 8–12 weeks were taken to reach the significant level related to the preventive effect (27, 37).

Folate plays a vital role in DNA synthesis and methylation *via* the metabolism of nucleotides, serving as the catalyst for essential biochemical processes, which embodies its importance in life progression (38). The deficient supply of folic acid is disruptive to human metabolic functions and contributes to some diseases, including cancer, geriatric disease, congenital defects, and inflammatory disorders (37–41). A population-based study observed that mothers with fortified folic acid before pregnancy had a decreased risk of CHD, and when compared with the middle quartiles of folic acid intake, low folic acid intake during pregnancy was related to an increased risk of CHD (3), which was compatible with the above finding. In China, the status of folic acid intake among women of childbearing age was unsatisfactory, and there were areas with a high prevalence of folic acid deficiency-related disease (42). As mentioned before, a time interval was required to reach an effective folic acid concentration, and our results emphasized that folic acid supplements should be applied in reproductive-age women as early as possible to avoid missing the critical period for the prevention of CHD.

In the present study, we analyzed the association between *MTHFR* genetic polymorphisms and the risk of CHD. After adjusted potential confounders, some genetic variants of the *MTHFR* gene, including rs2066470, rs1801133, and rs1801131 were explored to be significantly associated with an increased risk of CHD based on the Han Chinese population. The knowledge from the public database and kinds of literature explicitly uncover these SNPs are significant for coding. Notably, associations of genetic polymorphisms rs1801133 and rs1801131 at the *MTHFR* gene with the risk of CHD have been fully discussed despite the existence of dissenting findings (21, 23, 43–45). This study creatively analyzed eight SNPs of the *MTHFR* gene and probed the correlation of these SNPs with the development of CHD, which could provide a revealing insight into *MTHFR* genetic mutations correlated with the CHD risk.

The *MTHFR*, catalyzing the transformation of the 5,10-methylenetetrahydrofolate into the 5-methyltetrahydrofolate, gives a methyl which plays a key role in the process of homocysteine into methionine. It has been generally acknowledged that the *MTHFR* takes a significant part in the metabolism of folate and homocysteine (7, 8). What is intelligible is that genetic polymorphisms in the *MTHFR* gene may have an association with the development of CHD. Most previous studies focused on only 1-2 loci (i.e., rs1801133 and rs1801131); one resulted in the conversion of the amino acid alanine to valine (missense variants, A222V) and the other rendered a Glu-Ala change with decreased enzyme activity (46, 47), which was associated with the abnormal metabolism of homocysteine giving rise to hyperhomocysteinemia. In a word, it could reasonably deduce that aberrant expression or genetic mutations of the *MTHFR* gene may have a varied effect on the process of folate utilization, which could lead to the accumulation of homocysteine. Some studies provided definite evidence that genetic polymorphisms of the *MTHFR* gene increased homocysteine levels and were associated with the status of low folate (10, 44), which partly verified our presumption. The ponderable of the potential mechanism between the *MTHFR* gene and the CHD risk awaits further exploration.

We examined the different degrees of interactions between *MTHFR* genetic polymorphisms with maternal folic acid intake, which were the attractive findings of the present study. The study provided impressive evidence that the risk of CHD in offspring with variant genotypes whose mothers supplied folic acid, was significantly decreased when compared with children with mutant genotypes whose mothers did not fortify folic acid. In fact, few studies have been conducted to focus on this topic, and a previous study focused on the interplay between maternal *MTHFR* genetic variations and folic acid use on the occurrence of CHD in offspring (48). Our findings indicated that maternal folic acid supplements may partly modify the risk of CHD due to *MTHFR* genetic mutations. Considering the availability of the enzyme to folate metabolism, current folate fortification for women of childbearing age could need to have an extension for the prevention of CHD. Due to the poor understanding of the mechanisms underlying the regulation of embryonic cardiac progress, future research needs further exploration on this topic.

The limitations of the study need to be tackled. First, considering the source of samples, decided mainly by the respondents' numbers, the study did not select mothers who were abortive because of CHD, which could influence the representativeness of samples and extrapolation of the study findings. Second, though potential confounding factors were adjusted, it was ineludible that other confounding factors cannot be entirely controlled. Third, information on maternal exposure history was gathered through self-report, and therefore, recall bias was inevitable. Fourth, based on the limitation of the treatment time and the feature of folate, the internal exposure dose and the folate from natural sources and population food were not considered. Considering that the target population was restricted to Han Chinese individuals, more studies within other populations are needed. Fifth, the present study did not assess the risk of specific CHD subtypes limited by the sample size.

## Conclusion

The current findings observed that an association between maternal folic acid intake in the periconceptional period, the time when folic acid supplementation was started and the risk of CHD in offspring was found. In addition, polymorphisms of the *MTHFR* gene at rs2066470, rs1801133, and rs1801131 were significantly associated with the risk of CHD. Furthermore, our findings showed the different degrees of interaction between polymorphisms of the *MTHFR* gene and maternal folic acid intake. Considering the uncertainty of the mechanism and the limitation of the sample size, more studies therefore in different ethnic populations with a larger sample and prospective designs are warranted to verify our findings.

## Data availability statement

The datasets presented in this study can be found in online repositories. The names of the repository/repositories and accession number(s) can be found below: https://figshare.com/, https://doi.org/10.6084/m9.figshare.19729537.v1.

## Ethics statement

The studies involving human participants were reviewed and approved by the Ethics Committee of Xiangya School of Public Health Central South University (No. XYGW-2018-36). Written informed consent to participate in this study was provided by the participants' legal guardian/next of kin.

## Author contributions

SZ, JL, and YLi performed the experiments. JD and TW analyzed the data and statistical analyses. PZ and YLiu contributed reagents/material/analysis tools. TZ, XS, and JQ wrote the main manuscript text. JS, LC, JW, and MS collected references and managed data. All authors contributed to the article and approved the submitted version.

## Funding

This research was supported by the National Natural Science Foundation of China (Grant Numbers: 82073653 and 81803313), the China Postdoctoral Science Foundation (Grant Number: 2020M682644), the Science and Technology Planning Project of Guangdong Province (Grant Number: 2020A1414010152), the Hunan Provincial Science and Technology Talent Support Project (Grant Number: 2020TJ-N07), the Hunan Provincial Key Research and Development Program (Grant Number: 2018SK2063), and the Open Project from NHC Key Laboratory of Birth Defect for Research and Prevention (Grant Number: KF2020006).

## Conflict of interest

The authors declare that the research was conducted in the absence of any commercial or financial relationships that could be construed as a potential conflict of interest.

## Publisher's note

All claims expressed in this article are solely those of the authors and do not necessarily represent those of their affiliated organizations, or those of the publisher, the editors and the reviewers. Any product that may be evaluated in this article, or claim that may be made by its manufacturer, is not guaranteed or endorsed by the publisher.
