# Supplementary material for: Association of methylenetetrahydrofolate reductase gene polymorphisms and maternal folic acid use with the risk of congenital heart disease
Source: Front Pediatr. 2022 Sep 8;10:939119. doi: 10.3389/fped.2022.939119 (PMC9492935; doi:10.3389/fped.2022.939119)
Supplement: Supplementary file 1 [file Table_1.DOCX]

**Supplement Table S1 Comparison of baseline characteristics based on the use status of folic acid supplement in the control group**

| Variables | Folic acid supplement (yes) | Folic acid supplement (no) | Univariable analysis |
| --- | --- | --- | --- |
| Demographic characteristics |  |  |  |
| Gender |  |  | *χ²*=0.023, *P*=0.878 |
| Male | 376 (65.5%) | 28 (65.1%) |  |
| Female | 198 (34.5%) | 15 (34.9%) |  |
| Maternal age (≤35) | 518 (90.2%) | 35 (81.4%) | *χ²=*2.036*, P=*0.154 |
| Maternal education status |  |  |  |
| Less than primary or primary | 7 (1.2%) | 1 (2.3%） | *Z*=0.981, *P*=0.291^*^ |
| Junior high school | 97 (16.9%) | 16 (37.2%） |  |
| Senior middle school | 196 (34.1%) | 21 (48.8%） |  |
| College or above | 274 (47.7%) | 5 (11.7%） |  |
| Residence location (rural areas) | 318 (55.4%) | 21 (48.8%） | *χ²*=0.461, *P*=0.497 |
| Family history |  |  |  |
| Consanguineous marriages (yes) | 3 (0.5%) | 0 (0%) | *χ²*=0.221, *P*=0.639 |
| Adverse pregnancy history before this pregnancy | | |  |
| Spontaneous abortion (yes) | 53 (9.7%) | 7 (16.3%) | *χ²*=2.460, *P*=0.117 |
| Induced abortion or labor (yes) | 174 (30.3%) | 14 (32.6%) | *χ²*=0.168, *P*=0.682 |
| Fetal death or stillbirth (yes) | 2 (0.3%) | 0 (0%) | *χ²*=0.147, *P*=0.702 |
| Premature delivery (yes) | 6 (1.0%) | 0 (0%) | *χ²*=0.443, *P*=0.506 |
| Low birth weight (yes) | 3 (0.5%) | 0 (0%) | *χ²*=0.221, *P*=0.639 |
| Neonatal death (yes) | 0 | 0 (0%) | - |
| Ectopic pregnancy(yes) | 16 (2.0%) | 0 (0%) | *χ²*=1.202, *P*=0.273 |
| Congenital malformation(yes) | 2 (0.3%) | 0 (0%) | *χ²*=0.147, *P*=0.702 |
| Hypertension of pregnancy (yes) | 9 (1.6%) | 0 (0%) | *χ²*=0.668, *P*=0.414 |
| Gestational diabetes mellitus (yes) | 15 (2.6%) | 2 (4.7%) | *χ²*=0.673, *P*=0.412 |
| Personal lifestyle in the 3 months before this pregnancy | | |  |
| Active smoking | 10 (1.7%) | 3 (7.0%) | *χ²*=2.489, *P*=0.110 |
| Positive smoking | 213 (37.1%) | 14 (32.6%) | *χ²*=0.240, *P*=0.624 |
| Drinking alcohol | 43 (7.5%) | 0 (0%) | *χ²*=3.382, *P*=0.066 |
| Exposure to environmental risk factors in the 3 months before this pregnancy | | | |
| Harmful chemical | 40 (7.0%) | 7 (16.3%) | *χ²*=3.691, *p*=0.059 |
