# Supplementary material for: Association of methylenetetrahydrofolate reductase gene polymorphisms and maternal folic acid use with the risk of congenital heart disease
Source: Front Pediatr. 2022 Sep 8;10:939119. doi: 10.3389/fped.2022.939119 (PMC9492935; doi:10.3389/fped.2022.939119)
Supplement: Supplementary file 2 [file Table_2.DOCX]

**Supplement Table S2 Frequencies of *MTHFR* genotype and *P* values of HWE test**

| SNPs | Major allele | Minor allele | Type | Group | Allele frequency n (%) | |  | HWE test *P* |
| --- | --- | --- | --- | --- | --- | --- | --- | --- |
|  |  |  |  |  | Major allele | Minor allele |  |  |
| rs2274976 | C | T | Missense | Control | 1129 (91.5%) | 105 (8.5%) |  | 0.1902 |
|  |  |  |  | Case | 1036 (87.5%) | 148 (12.5%) |  |  |
| rs4846052 | C | T | Intron | Control | 1109 (89.9%) | 125 (10.1%) |  | 0.1047 |
|  |  |  |  | Case | 1016 (85.8%) | 168 (14.2%) |  |  |
| rs7525338 | C | T | Intron | Control | 1232 (99.8%) | 2(0.2%) |  | 0.9678 |
|  |  |  |  | Case | 1184 (100%) | 0 (0%) |  |  |
| rs4846051 | A | G | Missense | Control | 1232 (99.8%) | 2(0.2%) |  | 0.9678 |
|  |  |  |  | Case | 184 (100%) | 0 (0%) |  |  |
| rs1476413 | C | T | Intron | Control | 1035 (83.9%) | 199 (16.1%) |  | 0.3792 |
|  |  |  |  | Case | 904 (76.4%) | 280 (23.6%) |  |  |
| rs2066470 | G | A | Synonymous | Control | 1122 (90.9%) | 112 (9.1%) |  | 0.3496 |
|  |  |  |  | Case | 987 (83.4%) | 197 (16.6%) |  |  |
| rs1801133 | G | A | Missense | Control | 841 (68.2%) | 393 (31.8%) |  | 0.5066 |
|  |  |  |  | Case | 759 (64.1%) | 425 (35.9%) |  |  |
| rs1801131 | T | G | Missense | Control | 1060 (85.9%) | 174 (14.1%) |  | 0.5647 |
|  |  |  |  | Case | 886 (74.8%) | 298 (25.2%) |  |  |

HWE Hardy–Weinberg equilibrium, *MTHFR* Methylenetetrahydrofolate reductase, SNP single nucleotide polymorphism， GMS = genetic model selection
