# Supplementary material for: Association of methylenetetrahydrofolate reductase gene polymorphisms and maternal folic acid use with the risk of congenital heart disease
Source: Front Pediatr. 2022 Sep 8;10:939119. doi: 10.3389/fped.2022.939119 (PMC9492935; doi:10.3389/fped.2022.939119)
Supplement: Supplementary file 3 [file Table_3.DOCX]

**Supplement Table S3 The genetic model selection of SNPs at *MTHFR* gene**

| SNPs | Group |  | Genotype frequency n (%) | | | *Z*_HWDTT\|_ | GMS |
| --- | --- | --- | --- | --- | --- | --- | --- |
|  |  |  | AA | AB | BB |  |  |
| rs2274976 | Control |  | 519 (84.1%) | 91 (14.7%) | 7 (1.1%) | -0.213 | Additive |
|  | Case |  | 455 (76.9%) | 126 (21.3%) | 11 (1.9%) |  |  |
| rs4846052 | Control |  | 502 (81.4%) | 105 (17.0%) | 10 (1.6%) | 2.085 | Dominant |
|  | Case |  | 447 (75.5%) | 122 (20.6%) | 23 (3.9%) |  |  |
| rs1476413 | Control |  | 437 (70.8%) | 161 (26.1%) | 19 (3.1%) | -0.195 | Additive |
|  | Case |  | 349 (59.0%) | 206 (34.8%) | 37 (6.3%) |  |  |
| rs2066470 | Control |  | 512 (83.0%) | 98 (15.9%) | 7 (1.1%) | 2.574 | Dominant |
|  | Case |  | 423 (71.5%) | 141 (23.8%) | 28 (4.7%) |  |  |
| rs1801133 | Control |  | 283 (45.9%) | 275 (44.6%) | 59 (9.6%) | 1.464 | Additive |
|  | Case |  | 251 (42.4%) | 257 (43.4%) | 84 (14.2%) |  |  |
| rs1801131 | Control |  | 457 (74.1%) | 146 (23.7%) | 14 (2.3%) | -0.003 | Additive |
|  | Case |  | 333 (56.3%) | 220 (37.2%) | 39 (6.6%) |  |  |

SNP single nucleotide polymorphism, *MTHFR* Methylenetetrahydrofolate reductase, GMS = genetic model selection
