# Supplementary figures and images for: Association of methylenetetrahydrofolate reductase gene polymorphisms and maternal folic acid use with the risk of congenital heart disease
Source: Front Pediatr. 2022 Sep 8;10:939119. doi: 10.3389/fped.2022.939119 (PMC9492935; doi:10.3389/fped.2022.939119)

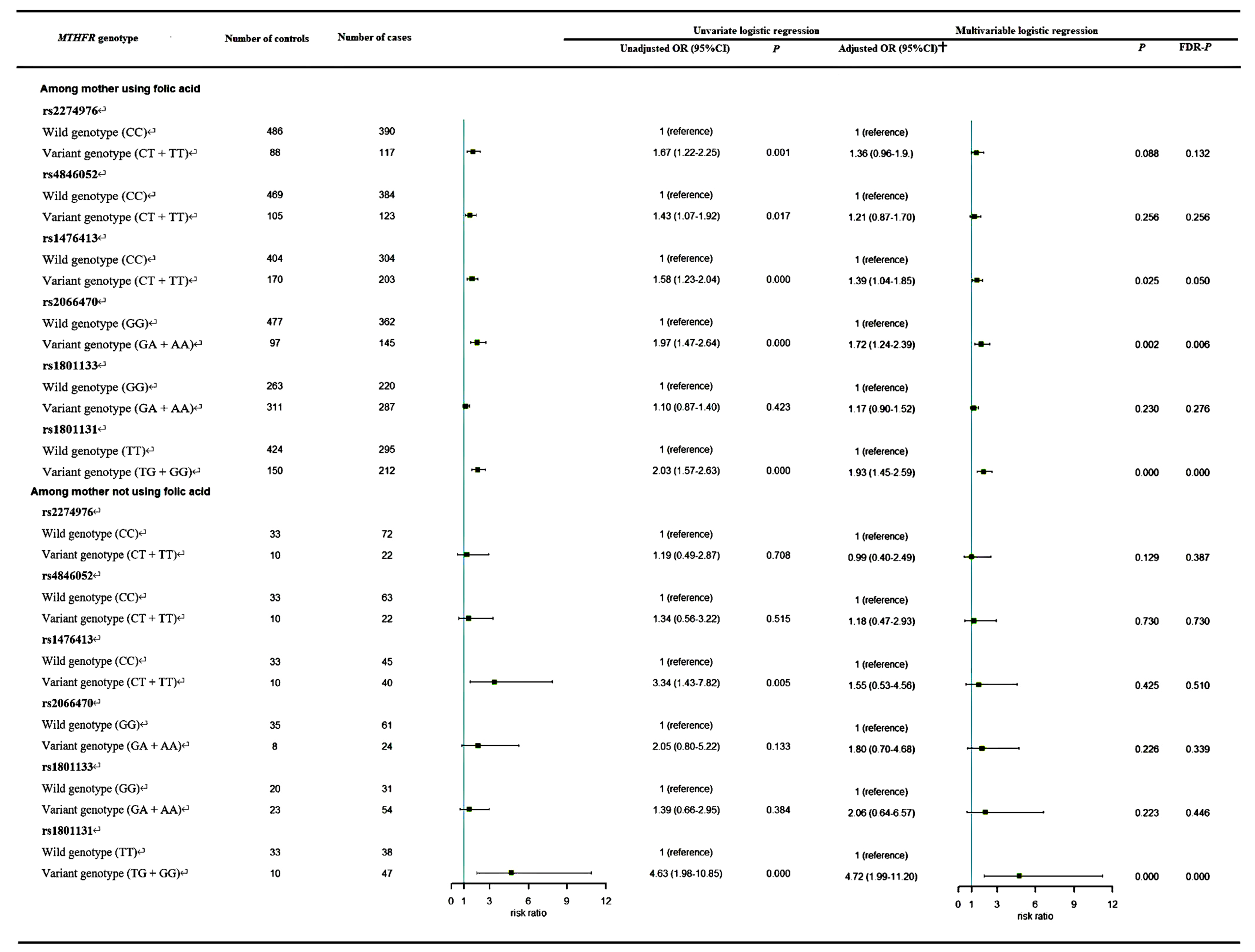

Supplement: Supplementary file 4 [file Image_1.TIF]
